# Cell type-dependent gene regulation by Staufen2 in conjunction with Upf1

**DOI:** 10.1186/1471-2199-12-48

**Published:** 2011-11-16

**Authors:** Takashi Miki, Yasunao Kamikawa, Sadamu Kurono, Yuka Kaneko, Jun Katahira, Yoshihiro Yoneda

**Affiliations:** 1 Department of Frontier Bioscience, Graduate School of Frontier Biosciences, Osaka University, Yamadaoka, Suita, Osaka, Japan; 2 Joint Research Laboratory of Molecular Signature Analysis, Division of Health Sciences, Graduate School of Medicine, Osaka University, Suita, Osaka, Japan; 3 Laboratory Chemicals Division, Wako Pure Chemical Industries, Ltd., Chuo, Osaka japan; 4 Department of Biochemistry, Graduate School of Medicine, Osaka University, Yamadaoka, Suita, Osaka, Japan

## Abstract

**Background:**

Staufen2 (Stau2), a double-stranded RNA-binding protein, is a component of neuronal RNA granules, which are dendritic mRNA transport machines. Although Stau2 is thought to be involved in the dendritic targeting of several mRNAs in neurons, the mechanism whereby Stau2 regulates these mRNAs is unknown. To elucidate the functions of Stau2, we screened for novel binding partners by affinity purification of GST-tagged Stau2 from 293F cells.

**Results:**

Three RNA helicases, RNA helicase A, Upf1 and Mov10, were identified in Stau2-containing complexes. We focused our studies on Upf1, a key player in nonsense-mediated mRNA decay. Stau2 was found to bind directly to Upf1 in an RNA-independent manner *in vitro*. Tethering Stau2 to the 3'-untranslated region (UTR) of a reporter gene had little effect on its expression in HeLa cells. In contrast, when the same tethering assay was performed in 293F cells, we observed an increase in reporter protein levels. This upregulation of protein expression by Stau2 turned out to be dependent on Upf1. Moreover, we found that in 293F cells, Stau2 upregulates the reporter mRNA level in an Upf1-independent manner.

**Conclusions:**

These results indicate that the recruitment of Stau2 alone or in combination with Upf1 differentially affects the fate of mRNAs. Moreover, the results suggest that Stau2-mediated fate determination could be executed in a cell type-specific manner.

## Background

Gene expression is regulated in various ways throughout the process of mRNA metabolism. In the nucleus, newly transcribed mRNA precursors undergo maturation via processing steps, such as 5' end capping, splicing, 3' end cleavage and polyadenylation. After being exported to the cytoplasm, the expression of mRNAs is further modulated by the regulation of their localization, stability and rate of translation.

In polarized cells, some mRNAs are localized at specific sites in the cytoplasm and are translated locally, thus enabling the spatiotemporal regulation of protein expression. In neurons, for example, several mRNAs that harbor localization signals, which are often detected at their 3'-untranslated regions (UTRs), are selectively transported to dendrites, while most mRNAs remain in the soma [1]. Neuronal RNA granules are dendritic mRNA transport machines that contain ribosomes, RNA-associated proteins and several translation factors, and they are transported by KIF5 along microtubules [2,3]. During cytoplasmic transport, mRNAs are considered to be translationally dormant because RNA granules lack tRNAs and other factors required to initiate translation [2].

Staufen2 (Stau2), a mammalian ortholog of the Staufen protein in *Drosophila melanogaster*, is a double-stranded RNA (dsRNA)-binding protein. Stau2 is expressed strongly in the brain and moderately in the heart [4,5]. In neurons, Stau2 is localized in the somatodendritic compartment and associates with RNA granules [4,6]. The overexpression of Stau2 in neurons increases the amount of poly (A)^+ ^mRNAs in dendrites [6]. The depletion of Stau2 in mature hippocampal neurons by RNA interference (RNAi) reduces the number of dendritic spines, suggesting that Stau2 regulates the targeting, translation and/or stabilization of the mRNAs involved in spine morphogenesis [7]. Although interactions of Stau2 with the mRNA binding proteins Tap and the Y14-Magoh heterodimer [5] have been reported, little is currently known concerning the functions of Stau2 in mRNA regulation. There are four splicing variants (Stau2^52^, Stau2^56^, Stau2^59 ^and Stau2^62^) of Stau2 [[8] and Additional File 1 Figure S1]. Among the Stau2 isoforms, nucleo-cytoplasmic shuttling proteins, Stau2^59 ^and Stau2^52 ^are exported from the nucleus through CRM1-dependent and -independent pathways, while Stau2^62 ^and Stau2^56 ^use only the CRM1-independent pathway [[8,9] and our unpublished data]. Beyond that, functional differences among the isoforms are not well known.

Upf1, an RNA helicase, was originally identified as an essential factor for nonsense- mediated mRNA decay (NMD) [10]. NMD is an mRNA surveillance mechanism that degrades mRNAs containing a premature termination codon to block the production of C-terminally-truncated proteins, which are potentially harmful to cells [11-13]. Tethering of Upf1 to the 3'-UTR of an mRNA, namely downstream of the termination codon, triggers mRNA decay in HeLa cells [14].

Staufen1 (Stau1), a paralog of Stau2, has been shown to induce the degradation of several mRNAs, such as ADP-ribosylation factor 1 (Arf1), in HeLa and C2C12 cells [15-17]. This mRNA decay mechanism resembles certain aspects of NMD (i.e., the essential recruitment of Upf1 to the target mRNA through protein-protein interactions) and is thus referred to as Stau1-mediated mRNA decay (SMD). However, whether Stau2 also triggers SMD remains unknown.

In this study, we identified Upf1 as a Stau2-interacting protein. We found that Stau2 interacts directly with Upf1 in an RNA-independent manner. Moreover, as expected from a previous report [5], we found that tethering Stau2 to the 3'-UTR of an mRNA has little effect on its expression in HeLa cells. In contrast, we found that tethering Stau2 to the 3'-UTR increases mRNA and protein levels in 293F cells. Intriguingly, Upf1 is only required for the upregulation of protein expression. These data suggest that Stau2 differentially regulates mRNA fate in a context-dependent manner.

## Results

### Identification of Upf1 as a Stau2-interacting partner

**Figure 1:**
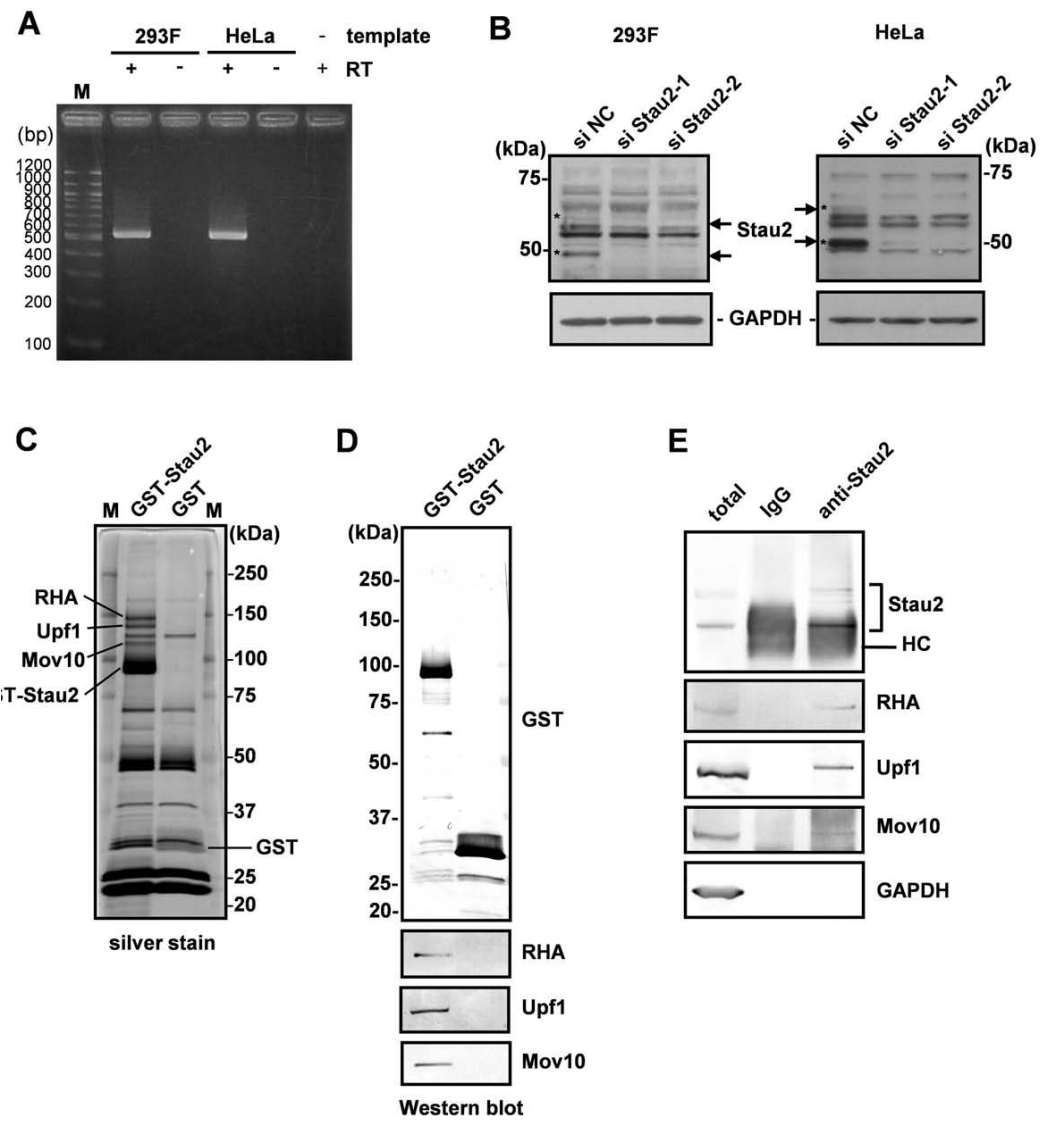
**Identification of Stau2-interacting proteins**. (A) A portion (nucleotides 396-907) of the human Stau2 cDNA (GenBank ID: NM_001164381) was amplified by RT-PCR from total RNA extracted from 293F or HeLa cells. The amplification was performed with (+) or without (-) a reverse transcription (RT) step. The products were analyzed by agarose gel electrophoresis followed by ethidium bromide staining. M; molecular weight marker. (B) 293F or HeLa cells were transfected with a negative control siRNA (siNC) or one of two different siRNAs to deplete Stau2 (siStau2-1 and siStau2-2). The cells were harvested at 48 h after siRNA transfection, lysed, and subjected to Western blotting using anti-Stau2 and anti-GAPDH antibodies. The positions of the Stau2 protein isoforms are indicated by asterisks. (C, D) GST-Stau2 and GST were precipitated from 293F-GST-Stau2 and 293F-GST cell extracts, respectively, with glutathione-Sepharose beads. After extensive washing, the bound proteins were eluted with reduced glutathione and subjected to SDS-PAGE. The protein bands were visualized by silver staining (C) and Western blotting using anti-GST, anti-RHA, anti-Upf1 and anti-Mov10 antibodies (D). The three specific bands migrating between 150 and 100 kDa were identified as RHA (142 kDa), Upf1 (124 kDa) and Mov10 (114 kDa) by mass spectrometry. M; molecular weight marker. (E) Immunoprecipitation from HeLa cell extracts was performed using a rabbit IgG or a rabbit anti-Stau2 antibody. Two percent of the total cell lysate and 10% of the precipitate were subjected to SDS-PAGE followed by Western blotting. HC; IgG heavy chain.

To identify proteins that interact with Stau2, we sought cell lines in which Stau2 was endogenously expressed and appeared to function. The expression of Stau2 mRNA in 293F and HeLa cells was confirmed by RT-PCR (Figure 1A). Furthermore, at least two among four splice isoforms of Stau2 [8] were detected by Western blotting in both 293F and HeLa cell extracts, and these became undetectable upon treatment with Stau2-targeting siRNAs (Figure 1B).

We established a 293F-derived cell line that stably expresses GST-fused Stau2 (293F-GST-Stau2) and purified Stau2-containing complexes from the cell extract by GST pull-down. The 293F cell line is a variant of the HEK 293 cell line, which can grow in FreeStyle 293 Expression Medium (Invitrogen) as suspension culture at high concentration (> 1 × 10^7^/ml). We took advantage of the characteristics for massive culture. Given that the HEK 293 cell line has been suspected to be of a neuronal cell lineage rather than a kidney epithelial cell lineage [18] and that Stau2 has been reported to be involved in mRNA regulation in neurons [4,6,7], interaction counterparts in the 293F cell line were also attractive targets. We chose Stau2^59 ^as bait from four Stau2 isoforms. As Stau2^59 ^is exported from the nucleus by two different pathways [9] and it has an extended C-terminal domain (Additional File 1 Figure S1), we expected that it might interact with a greater variety of proteins than other isoforms. Stau2 is a component of RNA granules, which are huge ribonucleoprotein (RNP) complexes containing a variety of proteins and RNAs [4,6]. Hence, it is likely that Stau2 associates with a variety of proteins, some of which indirectly interact via RNAs, thus making it difficult to identify direct binding partners of Stau2. To identify protein factors that directly bind to Stau2 but not proteins that indirectly interact via RNAs, we treated cell lysates with RNase A before purification. GST-Stau2-containing complexes were then isolated from the cell extract and separated by SDS-PAGE. As shown in Figure 1C, three prominent bands migrating between 150- and 100-kDa were detected only in the GST-Stau2 pull-down sample. These three bands were identified as RNA helicase A (RHA), Upf1 and Mov10 by mass spectrometric analysis (Figure 1C) and Western blotting (Figure 1D). These three proteins co-immunoprecipitated with endogenous Stau2 from HeLa cell lysates treated with RNase A (Figure 1E). Thus, three RNA helicases, RHA, Upf1 and Mov10, were identified as Stau2-interacting partners. As RNase A primarily digests single-stranded RNAs [19], we cannot exclude the possibility that some structured RNAs mediate the interaction between Stau2 and these RNA helicases.

### Stau2 interacts directly with Upf1

**Figure 2:**
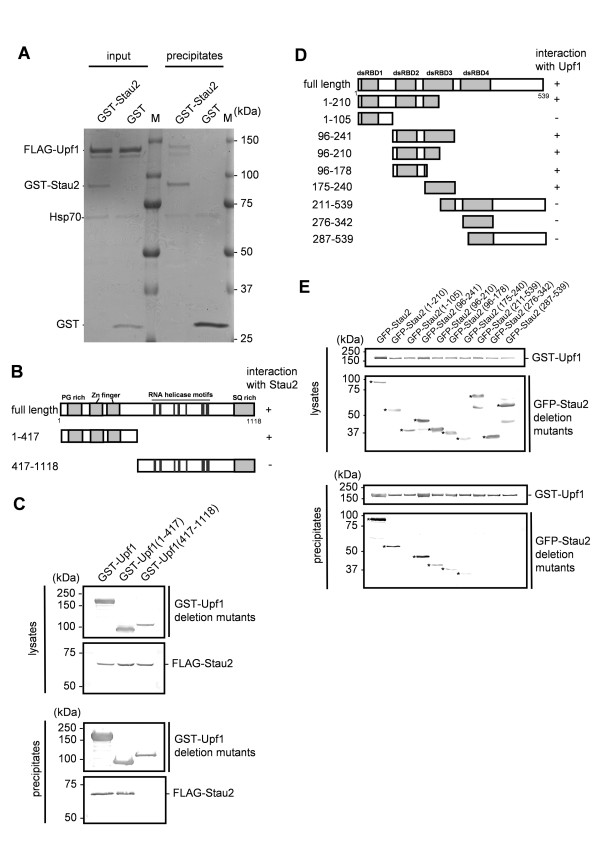
**Stau2 interacts directly with Upf1**. (A) Recombinant FLAG-Upf1 protein was mixed with either recombinant GST-Stau2 or GST, and the mixtures were incubated at 4 °C for 2 hr. The complexes were precipitated using glutathione-Sepharose beads. Ten percent of the total mixture and 30% of the precipitate were subjected to SDS-PAGE followed by CBB staining. Bands of contaminated Hsp70 proteins migrate between the 75- and 50-kDa markers. M; molecular weight marker. (B) Schematic representation of the Upf1 deletion mutants used. The numbers denote the amino acid positions relative to the N terminus of human Upf1. The gray boxes indicate a proline/glycine (PG)-rich region, zinc (Zn) finger domains and a serine/glutamine (SQ)-rich region. The dark gray boxes indicate conserved RNA helicase motifs. The ability to interact with Stau2 is indicated on the right. (C) Interaction between Upf1 deletion mutants and Stau2. Each Upf1 deletion mutant fused to GST was transiently co-expressed in 293F cells along with FLAG-Stau2. The cells were lysed at 24 h after transfection, and glutathione-Sepharose beads were used to pull down GST-Upf1 or its deletion mutants. The precipitates were analyzed by Western blotting using anti-GST or anti-Stau2 antibodies. Five percent of the total lysate (upper panels) and 30% of the precipitate (lower panels) were loaded. (D) Schematic representation of the Stau2 deletion mutants used. The numbers denote the amino acid positions relative to the N terminus of rat Stau2. The gray boxes indicate conserved dsRBDs. The ability to interact with Upf1 is indicated on the right. (E) Interaction between Upf1 and the Stau2 deletion mutants. GST-Upf1 was co-expressed in 293F cells with each GFP-fused Stau2 deletion mutant. The interaction was analyzed in the same way as in (C). The positions of the full-length and the deletion mutant proteins are indicated by asterisks.

Upf1 has been reported to interact with Stau1 to degrade a subset of mRNAs [15,16]. To address whether Stau2 has the equivalent function, we examined the interaction between Stau2 and Upf1. We purified recombinant Stau2 and Upf1 proteins from 293F cells and examined their interaction *in vitro*. As shown in Figure 2A, the recombinant Stau2 and Upf1 proteins interacted directly.

Upf1 consists of a proline/glycine (PG)-rich region at its N-terminus followed by two zinc (Zn)-finger domains, seven RNA helicase motifs and a serine/glutamine (SQ)-rich region at its C-terminus (Figure 2B) [20]. To determine which domain is involved in the interaction of Upf1 with Stau2, two deletion mutants of Upf1, Upf1 (1-417) and Upf1 (417-1118), were prepared (Figure 2B). Each GST-tagged Upf1 deletion mutant was co-expressed with FLAG-Stau2 in 293F cells and was pulled down from the lysates. The deletion mutant Upf1 (1-417), but not Upf1 (417-1118), co-precipitated with Stau2 (Figure 2C), indicating that the N-terminal region of Upf1, which contains the PG-rich region and the two Zn-finger domains, is sufficient for interaction with Stau2.

A similar method was used to identify the domain of Stau2 that interacts with Upf1. Combinations or individual dsRBDs tagged with green fluorescent protein (GFP) were co-expressed with GST-Upf1 in 293F cells, and their binding was tested by pull-down assays. Stau2 deletion mutants containing either dsRBD2 or dsRBD3 co-precipitated with Upf1 (Figure 2E and summarized in Figure 2D), suggesting that Stau2 interacts with Upf1 via dsRBD2 and/or dsRBD3, although we cannot exclude the possibility some of the small fragments were prevented from interacting with Upf1 by steric hindrance caused by GFP fusion.

### Tethering of Stau2 to the 3'-UTR of a reporter mRNA enhances protein expression in 293F cells

**Figure 3:**
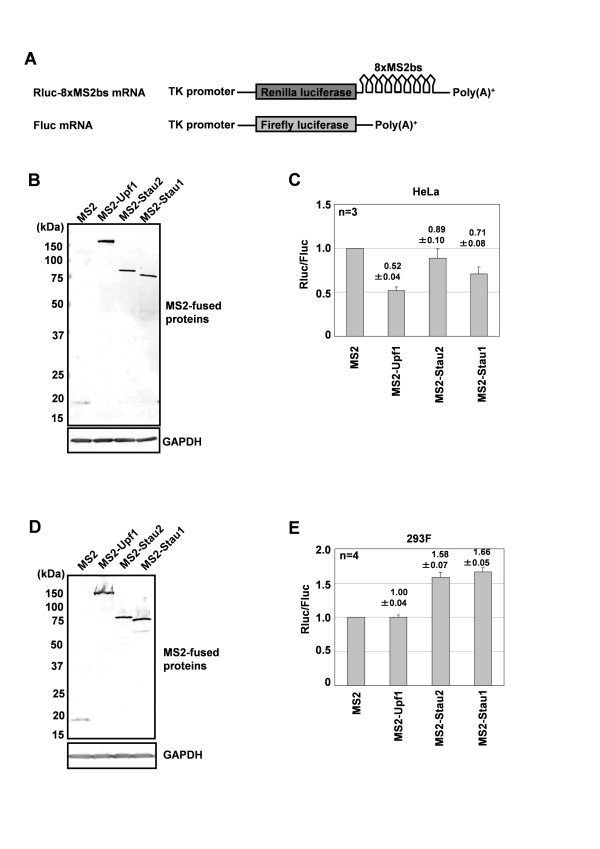
**Tethering of Stau2 to the 3'-UTR of a reporter mRNA enhances its protein expression in 293F cells**. (A) Schematic representations of the reporter constructs. The reporter Rluc-8xMS2bs consists of a cDNA encoding the Renilla luciferase (Rluc) protein with eight repeats of the MS2 binding site (8xMS2bs) in its 3'-UTR. The control plasmid Fluc contains a cDNA encoding the firefly luciferase (Fluc) protein. The expression of both mRNAs is under the control of the herpes simplex virus thymidine kinase (TK) promoter. (B-E) MS2-fusion proteins indicated below each panel were co-expressed in HeLa (B, C) or 293F cells (D, E) with Rluc-8xMS2bs and Fluc. At 48 h after transfection, the cells were harvested and subjected to Western blotting (B, D) or luciferase assay (C, E). (B, D) Western blotting was performed using an anti-HA antibody to detect MS2-fused proteins. (C, E) The expression of Rluc relative to that of Fluc was calculated for each transfection, and the normalized level of the MS2 transfection was defined as 1. Values are expressed as the means ± standard error of the mean (SEM) of four independent experiments.

Tethering either Upf1 [14] or Stau1 [15] to a 3'-UTR elicits mRNA decay in HeLa cells, whereas Stau2 has been reported to have little effect on the mRNA level [5]. It has also been reported that Stau2 and Upf1 associate with polysomes in neurons [4] and HeLa cells [21], respectively, suggesting that they play roles in translational regulation. Therefore, to address whether Stau2 and Upf1 cooperatively regulate gene expression, we examined whether Stau2 and Upf1 affect the expression of a luciferase gene when tethered to the 3'-UTR of its mRNA. We constructed a reporter plasmid containing a Renilla luciferase (Rluc) mRNA with eight repeats of the MS2 phage coat protein binding sequence in its 3'-UTR (Rluc-8xMS2bs) (Figure 3A). Because both Stau2 [5,22] and Upf1 [14,23] have been reported to interact with exon-exon junction complex (EJC) components, the reporter did not contain introns to minimize the effect of EJC formation. When MS2-fused Upf1, Stau2 or Stau1 was co-expressed in HeLa cells with the Rluc-8xMS2bs reporter together with Firefly luciferase (Fluc; which was used to normalize for transfection efficacy), Upf1 and Stau1 reduced the Rluc activity by approximately 50% and 30%, respectively, relative to the control (Figure 3B, C). On the other hand, Stau2 had little effect on reporter gene expression (Figure 3C). Thus, using our reporter system, we obtained results that are consistent with previously reported data in HeLa cells [5,14,15].

We next performed the same tethering assays using adherent culture of 293F cells. Unexpectedly, we found that expressing either MS2-Stau2 or MS2-Stau1 enhanced the expression of the reporter gene by approximately 60% or 70%, respectively, relative to the control (Figure 3D and Additional File 2 Figure S2). These results indicate that tethering Stau2 to the 3'-UTR of a reporter mRNA enhances protein expression in 293F cells, but not in HeLa cells, suggesting that Stau2 differentially regulates gene expression in a cell type-specific manner. Interestingly, the direct tethering of MS2-Upf1 had little effect on reporter expression in 293F cells (Figure 3E). This may indicate that the ability of Upf1 to trigger mRNA decay is attenuated in 293F cells.

### Stau2 tethered to the 3'-UTR of an mRNA enhances protein expression in a Upf1-dependent manner in 293F cells

**Figure 4:**
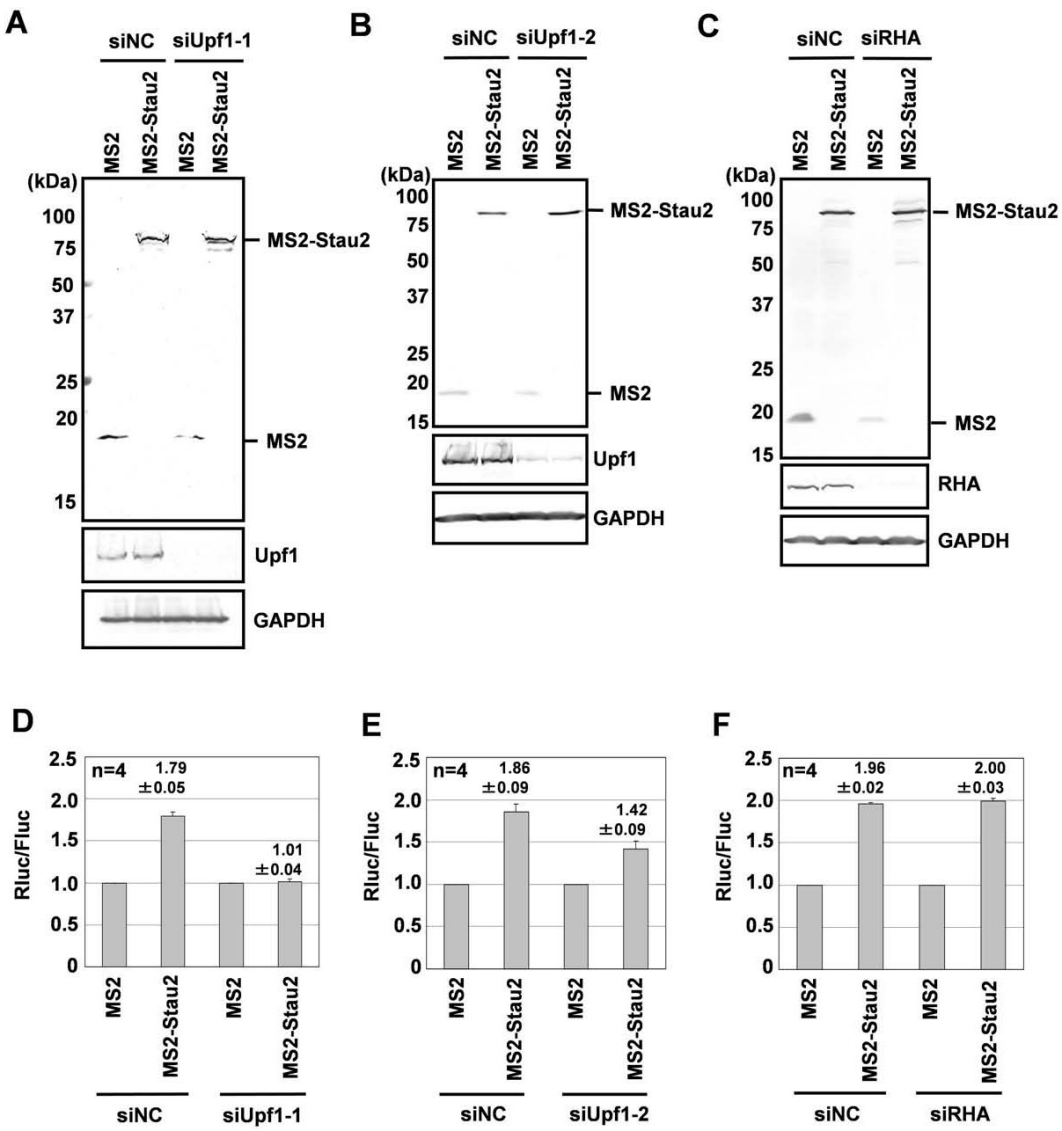
**The enhanced protein expression caused by tethering Stau2 to the 3'-UTR of an mRNA is mediated by Upf1**. 293F cells were transfected with an siRNA for Upf1 (siUpf1-1 or -2) or RHA (siRHA) or with a negative control siRNA (siNC). At 24 h after transfection, an expression plasmid for either MS2 or MS2-Stau2 was co-transfected with plasmids for Rluc-8xMS2bs and Fluc. At 48 h after plasmid transfection, the cells were harvested and subjected to Western blotting (A-C) or the luciferase assay (D-F). (A-C) Western blotting was performed using anti-HA, anti-Upf1, anti-RHA and anti-GAPDH antibodies. (D-F) The expression of Rluc relative to that of Fluc was calculated for each transfection, and the normalized level of the MS2 transfection was defined as 1. Values are expressed as the means ± SEM of four independent experiments.

We next investigated whether the enhancement of protein expression by Stau2 observed in 293F cells is dependent on Upf1. We depleted Upf1 from 293F cells by RNAi and examined the effect of Stau2 tethered to the 3'-UTR of Rluc-8xMS2bs. Stau2 failed to enhance the expression of the reporter gene in Upf1-depleted 293F cells (Figure 4A, D). Upf1 depletion using another siRNA also diminished the ability of Stau2 to enhance reporter gene expression (Figure 4B, E). Depletion of RHA, another RNA helicase that we identified as a Stau2-interacting partner (Figure 1B), did not affect the ability of Stau2 to enhance reporter gene expression (Figure 4C, F). These results indicate that Stau2 enhances protein expression by binding to the 3'-UTR of mRNAs in a Upf1-dependent manner.

### Stau2 tethered to the 3'-UTR increases mRNA levels independently of Upf1 in 293F cells

**Figure 5:**
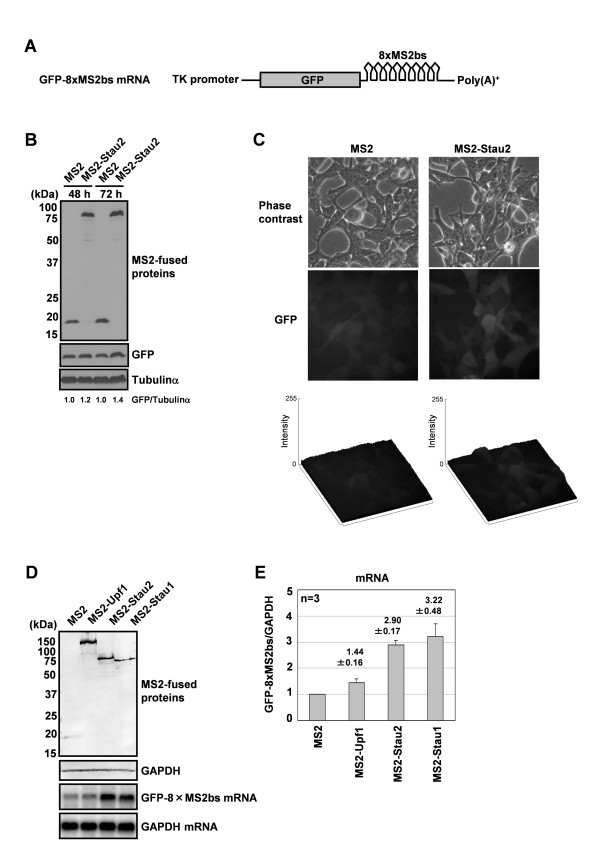
**Tethering Stau2 to the 3'-UTR increases mRNA levels in 293F cells**. (A) Schematic representation of the GFP-8xMS2bs mRNA. The 293F-GFP-8xMS2bs cell line stably expresses a reporter mRNA (driven by the TK promoter) encoding GFP with 8xMS2bs in its 3' UTR (B) Either MS2 or MS2-Stau2 was expressed in 293F-GFP-8xMS2bs cells. The cells were harvested at 48 h or 72 h after transfection and analyzed by Western blotting using anti-GFP and anti-Tubulin-α antibodies. The intensity of the GFP signal was normalized to that of Tubulin-α and is presented relative to MS2. (C) The expression of GFP in 293F-GFP-8xMS2bs cells transfected with an MS2 or MS2-Stau2 expression plasmid was observed by fluorescence microscopy at 72 h after transfection. The GFP signal intensities were analyzed and shown by surface plot. (D) MS2-fusion proteins were expressed in 293F-GFP-8xMS2bs cells. At 48 h after transfection, the cells were harvested and proteins and RNAs were extracted. The proteins were analyzed by Western blotting using anti-HA and anti-GAPDH antibodies. The GFP and GAPDH mRNAs were quantitated by Northern blotting using radiolabeled probes. (E) The GFP-8xMS2bs mRNA signal was normalized to that of GAPDH mRNA, and the normalized level of the MS2 transfection was defined as 1. Values are expressed as the means ± SEM of three independent experiments.

To determine whether the enhancement of protein expression by Stau2 is due to facilitated translation or an increase in mRNA stability, we examined the steady-state level of a reporter mRNA in the presence or absence of MS2-fusion proteins. We first examined mRNA expression using the Rluc-8xMS2bs reporter. However, we were not able to detect clear single bands suitable for quantitative determination (data not shown), as is often observed in transient expression experiments. To stabilize the expression of reporter mRNA and enable precise quantification, we established a 293F-derived cell line stably expressing a reporter mRNA encoding GFP with 8xMS2bs in its 3'-UTR (293F-GFP-8xMS2bs) (Figure 5A). This reporter system is not suitable for monitoring changes in protein levels because GFP has a long half-life (~26 h) in mammalian cells [24]. Nevertheless, we detected moderate increases in the level of GFP in 293F-GFP-8xMS2bs cells in which MS2-Stau2 had been expressed for 72 h by Western blot analysis (Figure 5B) and fluorescence microscopy (Figure 5C), supporting the results of the MS2-tethering assay using the Rluc-8xMS2bs reporter. Next, we expressed MS2-fused Upf1, Stau2 or Stau1 in 293F-GFP-8xMS2bs cells and quantitated the reporter mRNA by Northern blot analysis (Figure 5D). Both Stau2 and Stau1 increased the steady-state level of the reporter mRNA by approximately three-fold relative to the control, whereas Upf1 had little effect (Figure 5E). This result indicates that Stau2 and Stau1 stabilize mRNA by binding to the 3'-UTR in 293F cells.

**Figure 6:**
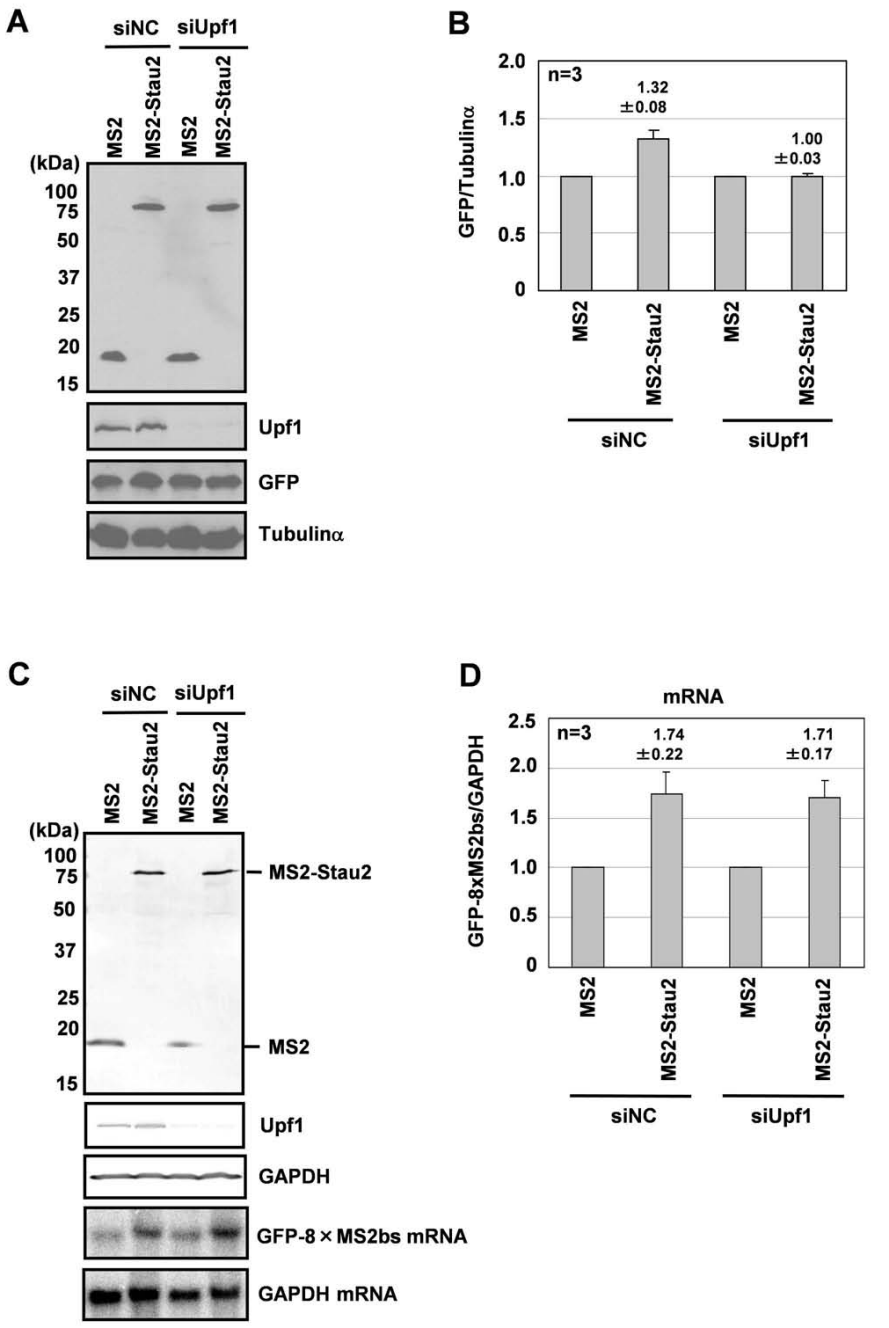
**Tethering Stau2 to the 3'-UTR increases mRNA levels in a Upf1-independent manner in 293F cells**. (A) 293F-GFP-8xMS2bs cells were transfected with siUpf1 or a siNC. At 24 h after transfection, the cells were transfected with an expression plasmid for either MS2 or MS2-Stau2. At 72 h after the plasmid transfection, the cells were harvested and analyzed by Western blotting. (B) The GFP signal was normalized to that of Tubulinα, and the normalized level of the MS2 transfection was defined as 1. Values are expressed as the means ± SEM of three independent experiments. (C) 293F-GFP-8xMS2bs cells were transfected with siUpf1 or siNC. At 24 h after transfection, the cells were transfected with an expression plasmid for either MS2 or MS2-Stau2. At 48 h after the plasmid transfection, the cells were harvested and analyzed by Western blotting and Northern blotting. (D) The GFP-8xMS2bs mRNA signal was normalized to that of GAPDH mRNA, and the normalized level of the MS2 transfection was defined as 1. Values are expressed as the means ± SEM of three independent experiments.

We next examined whether Upf1 is required for Stau2-mediated mRNA stabilization. Stau2 tethered to the 3'-UTR moderately increased the GFP protein level in control siRNA-treated cells, but not in Upf1-depleted cells (Figure 6A, B), supporting the results of the MS2-tethering assay using the Rluc-8xMS2bs reporter. Therefore, we then examined whether Upf1 is required for Stau2-mediated mRNA stabilization using this system. Stau2 tethered to the 3'-UTR stabilized the mRNA in Upf1-depleted cells and in control siRNA-treated cells (Figure 6C, D), indicating that when bound to a 3'-UTR, Stau2 can stabilize an mRNA independently of Upf1 in 293F cells.

## Discussion

In this study, we identified Upf1 as a Stau2-interacting partner by affinity chromatography (Figure 1). Stau2 was found to interact directly with Upf1 in an RNA-independent manner through its dsRBD2 and dsRBD3 domains (Figure 2). It has been shown that *in vitro*, dsRBD3 has strong dsRNA-binding activity, whereas dsRBD2 does not interact with dsRNA [4]. Although the possibility that the affinity between Stau2 and Upf1 might be regulated through the binding of dsRNA to dsRBD3 cannot be excluded, Stau2 can interact with Upf1 even in the absence of dsRNA. The dsRBD2 and dsRBD3 domains are conserved among all four Stau2 isoforms (Additional File 1 Figure S1), it is less likely that interaction with Upf1 is specific to Stau2^59^, which we used in this study. Given that a shorter isoform, probably Stau2^52^, is most abundantly expressed in 293F cells (Figure 1E), we assume that Stau2^52 ^is also involved in Upf1-dependent mRNA regulation in 293F cells. Stau1, a paralog of Stau2, has been reported to interact with Upf1 via a different domain that resides within dsRBD4 and the tubulin-binding domain [5]. However, our results (Figure 2B), together with a previous report [17], indicate that Upf1 uses the same domain (which resides in the N-terminus) to interact with both Stau1 and Stau2. Different binding modes of Stau2 and Stau1 to Upf1 might be responsible for the different results they produced in HeLa cells (see the discussion below).

It has been shown that Stau1 mediates mRNA decay by recruiting Upf1 to target mRNAs [15], although the target protein levels have not been examined. We found that tethering Stau2 to the 3'-UTR of a reporter mRNA has little effect on its protein level in HeLa cells (Figure 3C), consistent with the results reported by Monshausen *et al*. showing that Stau2 does not efficiently degrade mRNAs when tethered to their 3'-UTR in HeLa cells [5]. Thus, although we do not yet know why, Stau2 is not likely to trigger mRNA decay in HeLa cells, or at least it does so less efficiently than Stau1. The results from our immunoprecipitation experiments indicate that Stau2 interacts with Upf1 in HeLa cells (Figure 1E). Therefore, a possible explanation for these different effects is that the different domains that Stau2 and Stau1 use to interact with Upf1 might differentially regulate its activity.

In contrast to the data obtained in HeLa cells, we found that tethering Stau2 to the 3'-UTR of a target mRNA increased the target's mRNA (Figure 5E) and protein (Figure 3E, 5B) levels in 293F cells. Stau2 was able to stabilize mRNA, even in the Upf1-depleted condition (Figure 6D). Although we do not yet know the mechanism underlying this mRNA stabilization, Stau2 may recruit mRNA-associated proteins that protect the mRNA from degradation. Maher-Laporte *et al*. isolated Stau2-containing ribosome-free RNPs from the embryonic rat brain and identified poly (A)-binding protein cytoplasmic 1 (PABPC1) and Y box-binding protein 1 (YB1) in the particles [25]. Both PABPC1 [26,27] and YB1 [28,29] have been reported to be able to stabilize mRNA. Possibly, in 293F cells and in neurons, Stau2-bound mRNAs are stabilized in RNPs containing PABPC1 and YB1 during transport or until the time when the translation products are required.

In contrast to mRNA stabilization mediated by Stau2, its enhancement of protein expression is dependent on Upf1 in 293F cells (Figure 4, 6). This suggests that mRNAs stabilized by Stau2 are translationally dormant and that Stau2 can upregulate protein expression at the translational level in a Upf1-dependent manner, possibly via the recruitment of Upf1 to target mRNAs. As far as we know, this is the second report indicating that Upf1 functions positively in gene expression. Ajamian *et al*. showed that overexpressing Upf1 enhances, whereas depleting Upf1 reduces, HIV-1 expression at the RNA and protein levels in HeLa cells. This enhancement requires the ATPase activity of Upf1 but not its ability to interact with Upf2 [30]. It would be interesting to examine whether the ATPase activity of Upf1 is required for the upregulation of protein synthesis by the Stau2-Upf1 complex in 293F cells.

The results of our tethering assays indicate that Stau2, Stau1 and Upf1 regulate mRNA differently in HeLa and 293F cells (Figure 3). SMD, which has been reported to function in HeLa [15] and C2C12 [17] cells, is unlikely to operate in 293F cells. Although we do not yet know what causes this difference among cell types, this difference in SMD function is attributable, at least in part, to differences in the activity of Upf1 because Upf1 does not trigger mRNA decay in 293F cells when directly tethered to the 3'-UTR (Figure 5E). The ATPase activity of Upf1 is required for it to trigger mRNA decay [31]. A recent structural study by Chakrabarti *et al*. showed that binding to Upf2 induces a conformational change in Upf1 that turns on its ATPase activity [32]. The phosphorylation of Upf1 is also required for NMD [33,34], although its effects on Upf1's ATPase activity are still unknown. In addition, the efficiency of NMD has been reported to vary among different yeast strains [35], mammalian tissues [36,37] and even among different single cell-derived cell lines [38]. To test the possibility that the activity of Upf1 is differentially regulated in different cell lines, it would be tempting to look for differences in the association between Upf2 and Upf1 and in the phosphorylation of Upf1 in HeLa versus 293F cells.

In addition to Upf1, we identified two RNA helicases, RHA and Mov10, as Stau2-interacting partners (Figure 1), both of which are reportedly involved in translational regulation [39-41]. Interestingly, RHA was identified as a component of Stau1-containing RNPs in 293T cells [42], suggesting that RHA is a common interacting partner of Stau1 and Stau2. Given that RHA facilitates the translation of several mRNAs such as JUND mRNA thorough the structured post-transcriptional control element at their 5'-UTR [39], it would be interesting to examine the effects of tethering of Stau1 or Stau2 to a structured 5'-UTR on the mRNA translation.

## Conclusions

In this study, we examined the roles of Stau2 in mRNA regulation using artificial reporter systems. Our findings indicate that Stau2, when bound to a 3'-UTR, enhances mRNA translation and stabilizes the mRNA through Upf1-dependent and -independent mechanisms respectively in a cell-type specific manner. As far as we know, mRNAs that bind directly to Stau2 have not yet been identified. To understand mRNA regulation by Stau2 in more detail and to elucidate its physiological roles, mRNAs that are recognized specifically by Stau2 should be identified in future studies.

## Methods

### Cloning and plasmid construction

A mammalian expression vector, pCAGwTag, was constructed as follows: a neomycin resistance gene expression cassette isolated from the loxP-PGK-neoW vector [43] was inserted into a pBluescript SK(-) vector (Stratagene) that had been treated with SspI. The resulting plasmid was denoted as pBSPGKNeo. The pCAGGS vector [44] was digested with SalI and EcoRI to isolate a DNA fragment encoding the CAG promoter. A cDNA encoding glutathione S-transferase was amplified by PCR using pGEX6P3 (GE Healthcare) as a template. Both DNA fragments were inserted into the XhoI-BamHI site of pBSPGKNeo, and the resulting plasmid was designated as pBSpCAGGST. Double-stranded oligonucleotides encoding a 3xFLAG peptide tag sequence (DYKDHDGDYKDHDIDYKDDDDK) along with a DNA fragment encoding the rabbit β-globin polyadenylation signal, which had been isolated from pCAGGS by EcoRI and HindIII (blunt end) digestion, were inserted into pBSpCAGGST that had been linearized by treatment with MfeI and SacI (blunt end). The resulting vector, designated pCAGwTag, encodes glutathione S-transferase (GST) and 3xFLAG tags and contains the PreScission protease recognition sequence (LEVLFQGP) between the tags.

To construct the pCMV-MS2 vector, a cDNA encoding the MS2 phage coat protein in the pBC12/MS2 vector [45] was amplified by PCR using the following primers: forward, 5'-GATACCGGTCGCCACCATGAGCGCTTCTAACTTTACTCAG-3' and reverse, 5'-CATTGTACACTGCATAGTCCGGGACGTCATAGGGATAGCC-3'. The PCR product was digested with AgeI and BsrGI and cloned into the AgeI-BsrGI site of the pEGFP-C1 vector.

To construct the pRL-TK (-intron) vector, the pRL-TK vector (Promega) was digested with HindIII and NheI, blunted and self-ligated.

A cDNA encoding firefly luciferase was isolated from pGL3-basic (Promega) by digestion with NheI and BamHI. The cDNA fragment was ligated into the BamHI-NheI site of the pRL-TK vector. The resulting plasmid was designated pFL-TK.

To construct the pFL-TK (-intron) vector, pFL-TK was digested with HindIII, treated with Klenow fragment and then self-ligated. The pFL-TK (-intron) vector was used as a Fluc mRNA expression plasmid.

Rat Stau2 (GenBank ID: AY684789) was cloned as previously described [9]. To produce expression plasmids for GST-Stau2, FLAG-Stau2, GFP-Stau2 and MS2-Stau2, the cDNA encoding Stau2 was cloned into pCAGwTag, pCMV-FLAG, pEGFP-C1 (Clontech) and pCMV-MS2, respectively. The construction of GFP-Stau2 (1-210), GFP-Stau2 (1-105), GFP-Stau2 (96-210), GFP-Stau2 (175-240), GFP-Stau2 (211-539), GFP-Stau2 (276-342) and GFP-Stau2 (287-539) has been described previously [9]. A GFP-Stau2 (96-241) expression plasmid was constructed by inserting the BsrGI-AflII fragment of rat Stau2 cDNA, in which the AflII site had been blunted, into the BsrGI-SmaI site of the pEGFP-C2 vector. A GFP-Stau2 (96-178) expression plasmid was constructed by insertion of the NheI-HindIII fragment of GFP-Stau2 (96-210) into the NheI-HindIII site of the pEGFP-C2 vector.

A cDNA encoding human Upf1 (GenBank ID: NM_002911) was amplified from an EST clone (Open Biosystems Catalog No. MHS1010-9204820) that encodes the full length Upf1 cDNA by PCR using the following primers: forward, 5'-CTTAGATCTATGAGCGTGGAGGCGTACG-3' and reverse, 5'-GTAAGCTTCTCAAATCTCCCAGGTCC-3'. The PCR product was digested with BglII and HindIII and cloned into the pmRFP-C1 vector. To construct GST-Upf1 and MS2-Upf1 expression plasmids, the cDNA of Upf1 was cloned into pCAGwTag and pCMV-MS2, respectively. A GST-Upf1 (1-417) expression plasmid was constructed from GST-Upf1 by deleting the SalI fragment. A GST-Upf1 (417-1118) expression plasmid was constructed by inserting a SalI-EcoRI fragment isolated from GST-Upf1, in which the SalI site had been blunted, into the HindIII-EcoRI site of the pmRFP-C1 vector, in which the HindIII site had been blunted.

A cDNA encoding rat Stau1 (GenBank ID: NM_053436) was cloned from a rat brain cDNA library (Clontech) by PCR using the following primers: forward, 5'-GTCGAATTCATGTATAAGCCCGTGGACCCCCACTCTCGG-3' and reverse, 5'-GCTGGATCCCACCATGTCTTGGCCCACTGGAGTTATCAG-3'. The PCR product was digested with EcoRI and BamHI and cloned into pEGFP-C1. To construct an MS2-Stau1 expression vector, the Stau1 cDNA was cloned into the pCMV-MS2 vector.

To construct a renilla luciferase mRNA expression plasmid harboring the 8xMS2 binding site (8xMS2bs) at the 3'-UTR (pRL-TK (-intron)-8xMS2bs), a DNA fragment encoding 8xMS2bs was isolated from the RSV-β-gal-MS2bs-CaMKIIα3'UTR vector [46] by BamHI and BglII digestion, followed by T4 DNA polymerase treatment and ligated into a pRL-TK (-intron) vector that had been digested with XbaI and blunted.

A plasmid expressing a GFP mRNA harboring 8xMS2bs at its 3'-UTR (pTK-GFP-8xMS2bs) was constructed as follows: synthetic oligonucleotides encoding the FLAG epitope tag (top, AGCTACGCCACCATGGACTACAAGGACGACGATGACA and bottom, AGCTTGTCATCGTCGTCCTTGTAGTCCATGGTGGCGT) were annealed and ligated into the HindIII site of the pRL-TK vector (pRL-TK-FLAG). A DNA fragment that includes the TK promoter and the FLAG epitope was isolated from pRL-TK-FLAG by BglII and NheI digestion and ligated into the pEGFP-N1 vector from which the CMV promoter was deleted by AseI and NheI digestion (pTK-GFP). The 8xMS2bs fragment was isolated from the pRL-TK (-intron)-8XMS2bs vector by BamHI and NotI digestion followed by T4 DNA polymerase treatment and ligated into a pTK-GFP vector that had been treated with BsrGI-NotI and T4 DNA polymerase. Finally, the chimeric intron of the resulting plasmid was removed by digestion with BspMI and NheI.

### Antibodies

A rabbit polyclonal anti-rat Stau2 (rStau2) antibody was raised against the peptide sequence HLREKADNNQANPGSITQDC of rat Stau2. Immunization and affinity purification of the antibody were done at MBL Co., Ltd. A rabbit anti-human Stau2 (hStau2) antibody (RN013P, MBL), a rabbit anti-GST antibody (Z-5, Santa Cruz Biotechnology), a goat anti-Upf1 antibody (BL409G, Bethyl Laboratories), a rabbit anti-Mov10 antibody (A301-571A, Bethyl Laboratories), a mouse anti-HA antibody (HA-7, Sigma-Aldrich), a mouse anti-GAPDH antibody (6C5, Ambion), a rabbit anti-GFP antibody (A6455, Molecular Probes) and a mouse anti-Tubulin-α antibody (DM1A, Sigma-Aldrich) were purchased. Kotani *et al. *[47] generated the rat anti-RHA antibody (8E3).

### Cell culture

HeLa and 293F (Invitrogen) cells were cultured in Dulbecco's modified Eagle's medium (Sigma-Aldrich) supplemented with 10% fetal calf serum at 37 °C in a 5% CO_2 _atmosphere.

### Preparation of stable cell lines

The plasmids encoding GST-Stau2, GST or GFP-8xMS2bs were linearized by restriction enzyme digestion and transfected into 293F cells using Effectene (Qiagen). At 24 h after transfection, G418 (Gibco) was added at a final concentration of 0.8 mg/ml. After 3 days of incubation, the cells were re-plated, and individual clones were selected for their expression of GST-Stau2, GST, and GFP. These cell lines were designated 293F-GST-Stau2, 293F-GST, and 293F-GFP-8xMS2bs, respectively.

### RT-PCR

Total RNA was extracted from the 293F or HeLa cells using TRIzol reagent (Invitrogen) according to the manufacturer's protocol. To detect the expression of Stau2, the nucleic acid sequence 396-931 of human Stau2 cDNA (GenBank ID: NM_001164381) was amplified from 1 μg of total RNA by RT-PCR using the SuperScript III One-Step RT-PCR System (Invitrogen) according to the manufacturer's protocol. The following primers were used: forward, 5'-ACAAGCTGCTAGACACAATGCTGC-3', and reverse, 5'- GCATCACAAATTCTCGACGTCGAGG-3'. The PCR products were analyzed by agarose gel electrophoresis followed by ethidium bromide staining.

### RNA interference

Stealth siRNAs for the negative control (NC) and the depletion of Stau2 or RHA were obtained from Invitrogen: siNC, 5'-AGCUACACUAUCGAGCAAUUAACUU-3'; siStau2-1, 5'-GAGGAAUGCCUCGACGUCGAGAAUU-3'; siStau2-2, 5'-CCAAGCACUGCAGAAUGAACCUAUU-3'; siRHA, 5'-GGCAGCCAUGGAGGCUUUGGUUGUU-3'. For the depletion of Upf1, the following siRNAs were obtained from NipponEGT: siUpf1-1, 5'-GAUGCAGUUCCGCUCCAUU-d(TT)-3' [15]; siUpf1-2, 5'-CCAAGGCAUUGGCUUUUUAd(TT)-3'. The siRNAs were transfected into 293F or HeLa cells at a concentration of 1.6 nM using Lipofectamine RNAiMax reagent (Invitrogen) according to the manufacturer's protocol for reverse transfection. For experiments using exogenous protein expression, the culture medium was exchanged and the cells were transfected with expression plasmids for MS2 or MS2-Stau2 using Effectene at 24 h after siRNA transfection. At 48 h after plasmid transfection, the cells were harvested for luciferase assays, Northern blotting or Western blotting.

### Affinity chromatography

293F-GST-Stau2 or 293F-GST cells were cultured in suspension in 100 ml of FreeStyle 293 Expression Medium (Invitrogen) at 37 °C in a 5% CO_2 _atmosphere on an orbital shaker platform rotating at 125 rpm until the cell density reached 3 × 10^6^/ml. The cells were harvested, washed with PBS and then lysed in lysis buffer (20 mM Tris-HCl (pH 7.5), 150 mM NaCl, 1 mM DTT, 0.5% Tween 20, 20 μg/ml RNase A) for 15 min on ice. The cell lysates were centrifuged at 10,000 × g for 20 min to remove insoluble material. The supernatant was incubated with glutathione-Sepharose 4B beads (GE Healthcare) for 2 h at 4 °C with gentle agitation. The beads with bound proteins were isolated by centrifugation at 3,000 × g for 2 min. The beads were then washed five times with lysis buffer without RNase A. GST-Stau2 or GST proteins were eluted in 200 μl of elution buffer (100 mM Tris-HCl (pH 8.3), 100 mM NaCl, 2 mM DTT, 1 mM EDTA, 20 mM reduced glutathione) on ice for 15 min. The eluted proteins were separated by SDS-PAGE followed by silver staining. Each band was analyzed by mass spectrometry.

### Immunoprecipitation

HeLa cells (1 × 10^7^) were lysed in 1 ml of lysis buffer (20 mM Tris-HCl (pH7.5), 150 mM NaCl, 1 mM DTT, 0.5% Tween 20, 20 μg/ml RNase A) for 15 min on ice. The cell lysates were centrifuged at 10,000 × g for 20 min to remove insoluble material. The supernatant was incubated with rabbit IgG (10 μg) or a rabbit anti-hStau2 (10 μg) antibody for 1 h on ice followed by the addition of Protein G Sepharose 4 Fast Flow (GE Healthcare) and incubation at 4 °C with gentle agitation for 2 h. The beads with the bound proteins were isolated by centrifugation at 3,000 × g for 2 min. The beads were washed three times with 1 ml of lysis buffer without RNase A. The bound proteins were eluted in 100 μl of SDS sample buffer (63 mM Tris-HCl (pH 6.8), 5 mM DTT, 2% SDS, 5% sucrose, 0.01% Bromophenol Blue). Two percent of the total lysate and 10% of the precipitate were separated by SDS-PAGE followed by Western blotting.

### Recombinant protein production

Recombinant GST-Stau2 and GST proteins were purified from 293F-GST-Stau2 and 293F-GST cells, respectively. The cells were cultured in suspension in FreeStyle 293F Expression Medium at 37 °C in a 5% CO_2 _atmosphere on an orbital shaker platform rotating at 125 rpm. The cells were harvested, washed with PBS, and then lysed in lysis buffer (20 mM Tris-HCl (pH 7.5), 800 mM NaCl, 1 mM DTT, 0.5% Tween20, 20 μg/ml RNase A) for 15 min on ice. The cell lysates were centrifuged at 12,500 × g for 20 min to remove insoluble material. The supernatant was incubated with glutathione-Sepharose 4B beads for 2 h at 4 °C with gentle agitation. The beads with bound proteins were isolated by centrifugation at 3,000 × g for 2 min. The beads were washed six times with lysis buffer without RNase A. GST-Stau2 or GST protein was eluted in elution buffer (100 mM Tris-HCl (pH 8.3), 100 mM NaCl, 2 mM DTT, 1 mM EDTA, 20 mM reduced glutathione) on ice for 15 min. The solvent was then replaced by stock buffer (20 mM Tris-HCl (pH7.5), 150 mM NaCl, 1 mM DTT) using PD-10 Desalting Columns (GE Healthcare).

Recombinant FLAG-Upf1 protein was produced using the FreeStyle 293F Expression System (Invitrogen) according to the manufacturer's protocol. Briefly, GST-FLAG-Upf1 was transiently transfected into 293F cells. The cells were lysed, and GST-FLAG-Upf1 was purified using glutathione-Sepharose 4B beads as described above. The FLAG-Upf1 protein was eluted in stock buffer by PreScission protease digestion (GE Healthcare).

### In vitro binding assay

Recombinant FLAG-Upf1 protein (5 μg) was mixed with recombinant GST-Stau2 protein (1 μg) or GST protein (1 μg) in 800 μl of binding buffer (20 mM Tris-HCl (pH 7.5), 150 mM NaCl, 1 mM DTT, 0.05% Tween 20) at 4 °C for 2 hr with gentle agitation and then precipitated using glutathione-Sepharose 4B beads. The beads were washed five times with binding buffer. The bound proteins were eluted in 50 μl of sample buffer (60 mM Tris-HCl (pH 6.8), 1.7% SDS, 6% glycerol, 100 mM DTT, 0.002% bromophenol blue). Ten percent of the total cell extract and 30% of the precipitate were analyzed by SDS-PAGE followed by Coomassie brilliant blue (CBB) staining.

### In vivo binding assay

293F cells were cotransfected with the Stau2 and Upf1 expression vectors using Effectene (Qiagen) according to the manufacturer's protocol. At 24 h after transfection, the cells were harvested and lysed in 800 μl of lysis buffer (20 mM Tris-HCl (pH 7.5), 150 mM NaCl, 1 mM DTT, 0.5% Tween20, 20 μg/ml RNase A). The lysate was centrifuged at 12,500 × g for 20 min to remove insoluble material. The supernatant fraction was incubated with glutathione Sepharose beads for 2 h at 4 °C with gentle agitation. The beads were then washed five times with lysis buffer without RNase A. The bound proteins were eluted in 50 μl of sample buffer. Five percent of the total cell lysate and 30% of the precipitate were analyzed by SDS-PAGE and Western blotting.

### MS2 tethering assay

293F or HeLa cells cultured in 35-mm dishes were co-transfected with plasmids encoding Rluc-8xMS2bs (0.1 μg) and Fluc (0.1 μg) mRNAs along with a plasmid encoding an MS2-fused protein (0.2 μg) with or without a plasmid encoding a GST-fusion protein (0.4 μg). At 48 h after transfection, the cells were harvested and assayed for luciferase activity using the Dual-Luciferase Reporter Assay System (Promega) according to the manufacturer's protocol. The Rluc activity in each transfection was normalized to the co-expressed Fluc activity. At least three independent transfections were performed in each experiment.

293F-GFP-8xMS2bs cells were cultured in 60-mm dishes and transfected with a plasmid encoding an MS2-fusion protein (1.0 μg). At 48 h after transfection, total RNA was extracted from the cells using TRIzol reagent. Two micrograms of total RNA was separated on a 1.3% agarose-formaldehyde gel and transferred to a Hybond-N+ membrane (GE Healthcare). A template cDNA for GFP was isolated from the pEGFP-C1 vector by NheI-BsrGI digestion. A template cDNA for GAPDH was amplified by PCR using the following primers: forward, 5'-CAAGGTCATCCCTGAGCTGAAC-3' and reverse, 5'-GGGTCTACATGGCAACTGTGAG-3'. ^32^P-labeled probes were prepared using a random prime DNA labeling kit (Takara Biomedicals). Hybridization was carried out in Hybridization Solution (Nacalai tesque) according to the manufacturer's protocol. The membranes were exposed to an imaging plate (Fuji Film), and the signals were visualized using a Bio-Imaging analyzer (BAS-2500, Fuji Film). The intensity of the signals below saturation was measured by Scion Image (Scion Corporation). The signal intensity of GFP-8xMS2bs mRNA in each transfection was normalized to that of GAPDH mRNA.

### Fluorescence microscopy

At 72 h after transfection, 293F-GFP-8xMS2bs cells transiently expressing MS2 or MS2-Stau2 were observed using a Zeiss Axiovert 200 fluorescent microscope (Carl Zeiss). GFP signal intensities were analyzed by ImageJ software surface plot program.

## List of abbreviations

Stau2: Staufen2; dsRNA: double-stranded RNA; UTR: untranslated region; NMD: nonsense-mediated mRNA decay; RNAi: RNA interference; Stau1: Staufen1; Arf1: ADP-ribosylation factor 1; SMD: Stau1-mediated mRNA decay; GST: glutathione S-transferase; CBB: Coomassie brilliant blue; dsRBD: dsRNA-binding domain; RNP: ribonucleoprotein; PG: proline/glycine; Zn: Zinc; SQ: serine/glutamine; GFP: green fluorescence protein; Rluc: Renilla luciferase; Fluc: Firefly luciferase; MS2bs: MS2 coat protein binding sequence; EJC: exon-exon junction complex; TK: thymidine kinase.

## Authors' contributions

TM designed the experiments, acquired data and wrote the manuscript. YaK acquired data. SK and YuK performed mass spectrometry. JK designed the experiments and wrote the manuscript. YY wrote the manuscript. All authors read and approved the final manuscript.

## Supplementary Material

Additional file 1 **Schematic representation of Stau2 isoforms**. Stau2 has four splice variants, and they differ in N- and C-terminal sequences each other. Click here for file

Additional file 2 **Specificity validation of the MS2 tethering assay system**. To confirm that MS2-fusion proteins are specifically tethered to the MS2 binding sites at 3'-UTR of the reporter mRNA, we show that GST-Stau2 does not affect the activity of the reporter with MS2 binding sites and that MS2-Stau2 does not affect the activity of the reporter without MS2 binding sites. Click here for file
