# Supplementary material for: Cell type-dependent gene regulation by Staufen2 in conjunction with Upf1
Source: BMC Mol Biol. 2011 Nov 16;12:48. doi: 10.1186/1471-2199-12-48 (PMC3226675; doi:10.1186/1471-2199-12-48)
Supplement: Additional file 1 — Schematic representation of Stau2 isoforms. Stau2 has four splice variants, and they differ in N- and C-terminal sequences each other. [file 1471-2199-12-48-S1.PDF]

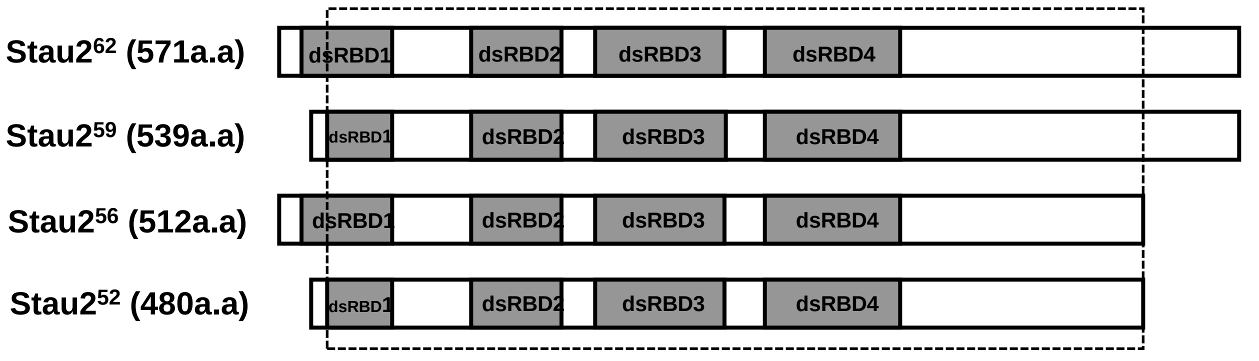

Figure S1–Schematic representation of Stau2 isoforms

Stau2 has four splice isoforms, as denoted by molecular mass with number of amino acids (a.a). Dark grey boxes indicate the dsRBDs. A common region among the four Stau2 isoforms is surrounded by dashed line.
