# Supplementary material for: Cell type-dependent gene regulation by Staufen2 in conjunction with Upf1
Source: BMC Mol Biol. 2011 Nov 16;12:48. doi: 10.1186/1471-2199-12-48 (PMC3226675; doi:10.1186/1471-2199-12-48)
Supplement: Additional file 2 — Specificity validation of the MS2 tethering assay system. To confirm that MS2-fusion proteins are specifically tethered to the MS2 binding sites at 3'-UTR of the reporter mRNA, we show that GST-Stau2 does not affect the activity of the reporter with MS2 binding sites and that MS2-Stau2 does not affect the activity of the reporter without MS2 binding sites. [file 1471-2199-12-48-S2.PDF]

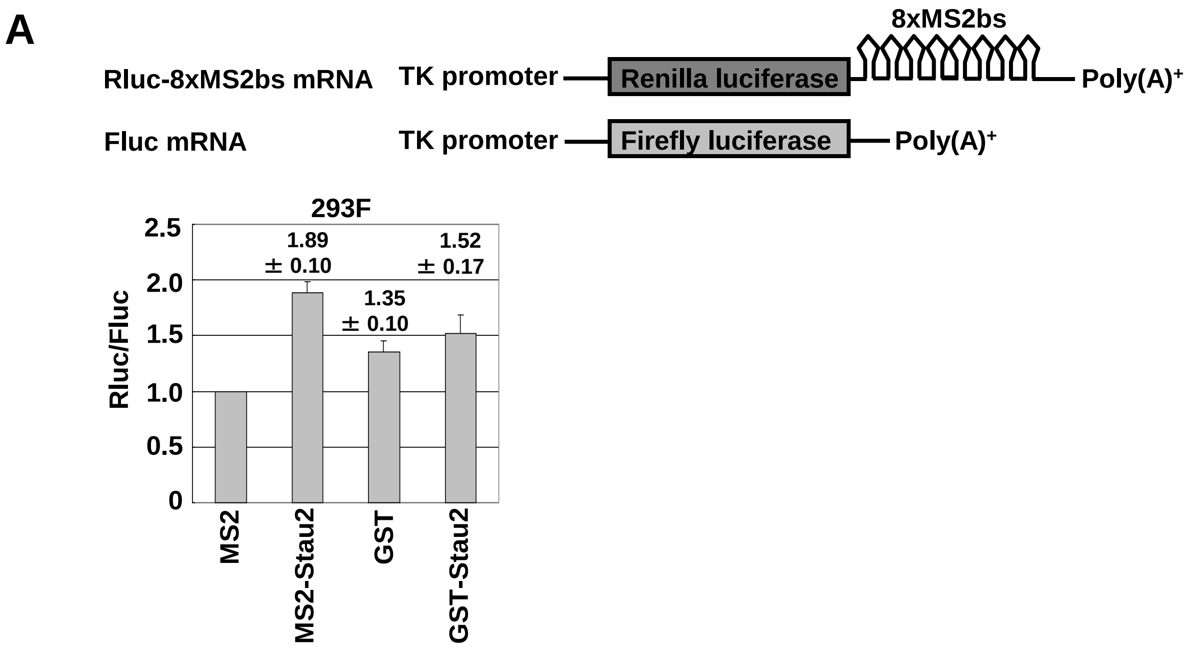

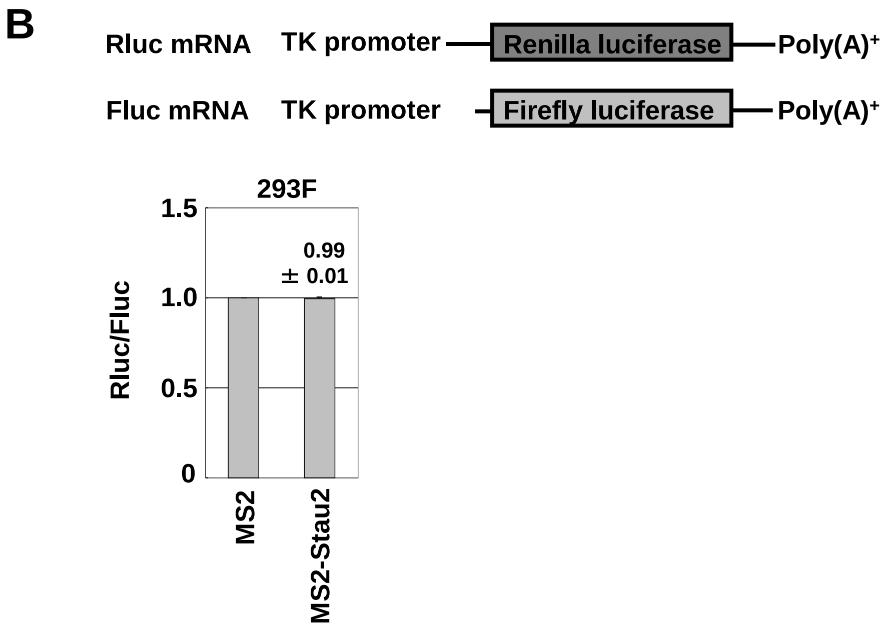

Figure S2 -Specificity validation of the MS2 tethering assay system
MS2-fusion proteins or GST-fugion proteins indicated below each panel were co-expressed with Rluc-8xMS2bs (A) or Rluc (B) together with Fluc in 293F cells. At 48 h after transfection, the cells were harvested and subjected to luciferase assay The expression of Rluc relative to that of Fluc was calculated for each transfection, and the normalized level of the MS2 transfection was defined as 1. Values are expressed as the means ±SEM of three independent experiments.
